# Assessment of *DPY19L2* Deletion in Familial and Non-Familial Individuals with Globozoospermia and *DPY19L2* Genotyping 

**DOI:** 10.22074/ijfs.2016.4910

**Published:** 2016-06-01

**Authors:** Parastoo Modarres, Somayeh Tanhaei, Marziyeh Tavalaee, Kamran Ghaedi, Mohammad Reza Deemeh, Mohammad Hossein Nasr-Esfahani

**Affiliations:** 1 Department of Cellular Biotechnology, Cell Science Research Center, Royan Institute for Biotechnology, ACECR, Isfahan, Iran; 2 Department of Reproductive Biotechnology, Reproductive Biomedicine Research Center, Royan Institute for Biotechnology, ACECR, Isfahan, Iran; 3 Department of Biology, Faculty of Sciences, University of Isfahan, Isfahan, Iran; 4 Isfahan Fertility and Infertility Center, Isfahan, Iran

**Keywords:** Gene Expression, Genotyping, Globozoospermia

## Abstract

**Background:**

Globozoospermia is a rare syndrome with an incidence of less than 0.1% among infertile men. Researchers have recently identified a large deletion, about 200 kbp, encompassing the whole length of *DPY19L2* or mutations in *SPATA16* and *PICK1* genes associated with globozoospermia. The aim of this study was to analyze the *DPY19L2* gene deletion using polymerase chain reaction technique for the exons 1, 48, 11 and 22 as well as break point (BP) “a” in globozoospermic men.

**Materials and Methods:**

In this experimental study, genome samples were collected from 27 men with globozoospermia (cases) and 36 fertile individuals (controls), and genomic analysis was carried out on each sample.

**Results:**

Deletion of *DPY19L2* gene accounted for 74% of individuals with globozoospermia. *DPY19L2* gene deletion was considered as the molecular pathogenic factor for the onset of globozoospermia in infertile men. By quantitative real-time polymerase chain reaction (qPCR), we genotyped *DPY19L2* deletion and identified carriers within the population.

**Conclusion:**

This technique may be considered as a method for family counseling and has the potential to be used as a pre-implantation genetic diagnosis, especially in ethnic community with high rate of consanguineous marriages.

## Introduction

Globozoospermia is a rare autosomal recessive genetic syndrome with an incidence of less than 0.1%. In this syndrome, due to defect in the process of acrosome biogenesis, the sperm contains a round head shape, con- sequently leading to no penetration into the oocyte during fertilization. Thus, direct intra- cytoplasmic sperm insemination (ICSI) along with artificial oocyte activation is the only so- lution to gain pregnancy at couples suffering this abnormality (1). Genetic pedigree assess- ment of these individuals indicates the con- genital origin of globozoospermia. To define molecular defects involved in this disorder, several autosomal genes have been identified in knockout mice models including: *Csnk2a2, Hrb, Gopc, Pick1, Gba2, Vps54, Zpbp1* and *Hsp90b1* (2-9). Defect of these genes in mouse models represented phenotypically similar abnormalities to human globozoospermia. However, among the aforementioned genes, only *PICK1* mutation was yet detected in hu- man. *PICK1* protein is involved in subcellular trafficking in brain, pancreas and testis. The respective gene is located on human chromosome 22, and contains 13 exons. In spermatogenesis, *PICK1* is involved in trafficking of pro-acrosomal vesicles from golgi apparatus to acrosome. Liu et al. (10) showed a ho- mozygous missense mutation (G198A) at the C-terminal domain of *PICK1* which disrupted *PvuII* site, culminating in formation of sperms with round head shape in human. Other human autosomal genes involved in globozoospermia are *SPATA16* and *DPY19L2* (11-13). *SPATA16* is a testis specific gene, translating a protein which is localized in the golgi apparatus and plays a role in the transportation of pro-acro- somal vesicles from golgi to the acrosome in the round and elongated spermatids (14). Dam et al. (11) found a homozygous sequence vari- ation in the last nucleotide of exon 4 (G848A) of this gene which impaired *NciI* or *HpaII* recognition site, in three infertile brothers of a Jewish family with globozoospermia.

However, the most likely considered gene to have a pivotal role in globozoospermia is *DPY19L2*. This gene is expressed primarily in spermatids with a specific localization limited to the inner nuclear membrane, facing the acroso- mal vesicle. Lack of the relevant protein causes instability of acrosome vesicles and thereby loss of acrosome (15). It has been demonstrated that complete deletion of *DPY19L2* by non-allelic homologous recombination (NAHR) results in globozoospermia (12, 13). Recent studies have revealed that *DPY19L2* gene function could be eliminated at nine possible breakpoints covering three regions, known as breakpoints “a, b and c” in two low copy flanking repeats (LCRs) of *DPY19L2* gene. High incidence (96.5%) of LCR sequences facilitates the occurrence of NAHR in this region (16).

Considering the role of aforementioned genes in globozoospermia and in line with our per- vious study (16), the aim of this study was to evaluate the prevalence of missense mutations, G848A, in exon 4 of *SPATA16* gene and G198A in exon 13 of *PICK1*, as well as *DPY19L2* de- letion in Iranian infertile individuals with globozoospermia referring to Isfahan fertility and infertility center (IFIC). Herein, we observed complete deletion of *DPY19L2* gene in 20 out of 27 globozoospermic individuals, but no mutation was detected in *SPATA16* or *PICK1* gene. We also performed quantitative real-time polymerase chain reaction (qPCR) assay to identify individuals with homo/hemizygous deletion of *DPY19L2* gene.

## Materials and Methods

### Mutational analysis of *SPATA16*, *PICK1* and *DPY19L2* genes

This experimental study was approved by Institutional Review Board (IRB) of Royan Institute. In this case-control study, 27 male with globozoospermia from Iranian population were contributed. An arbitrary number was assigned to each globozoospermic individual (G1 to G29), out of whole two individuals, G11 and G17, were omitted due to missing. We assessed the mutations for *SPATA16* and *PICK1* genes and provided pedigrees for two families with complete deletion of *DPY19L2* and one family with deletion of exon 5, 6 and 7 in *DPY19L2* gene.

In this process, blood samples were taken from 27 individuals, with globozoospermia with round-headed spermatozoa who referred to IFIC, as well as their family members after completing a consent form. Two out of 27 persons with more than 50% acrosomeless spermatozoa in their normal and round-headed sperm samples were considered to have partial globozoospermia, while the rest of individuals were suffering from total globozoospermia. Peripheral blood samples were also taken from 30 fertile men as well as the parents of three individuals with globozoospermia (G8, 14 and 21). In the sample group, except three brothers (G21, 22, and 23) and two cases of five (G5, 6, 20, 26, and 27) and two (G9 and 29) cousin subjects, the remaining 17 individuals with globozoospermia belonged to unrelated families (Table 1).

**Table 1:** Features of 27 individuals with globozoospermia


Patient	Type of globozoospermia		Consanguinity	Deficiency in *DPY19L2* gene				Reference
	Complete	Partial		No deletion	Complete deletion		Partial deletion (exous 5, 6, 7)	
					Unknown break point	Break point "a"		

G1, 4, 7, 12, 13, 15	✓		Non-familial		✓			(16)
G2, 8, 10, 14,16	✓		Non-familial			✓		(16)
G3, 18	✓		Non-familial	✓				(16)
G19		✓	Non-familial	✓				(16)
G5, 6, 20	✓		familial			✓		(16)
G9	✓		familial(G29)				✓	(16)
G21, 22, 23	✓		familial			✓		Current study
G24	✓		Non-familial	✓				Current study
G25, 28		✓	Non-familial	✓				Current study
G26, 27	✓		Familial (G5)			✓		Current study
G29	✓		Familial (G9)				✓	Current study


Genomic DNA was extracted from individuals’ peripheral blood samples using standard salting out procedure and kept at -20 C until usage (17). Specific primers for identification of G848A, in exon 4 of *SPATA16* gene and G198A in exon 13 of *PICK1* gene were designed by oligo7 primer designing software (Molecular Biology Insights, CO, USA) according to the respective sequences obtained from National Center for Biotechnology Information (NCBI) database, whereas primer sequences (Table 2) for assessment of *DPY19L2* deletion were ordered according to previous report (16). Missense mutations of *SPATA16* and *PICK1* genes were assessed using Restriction Fragment Length Polymorphism PCR (RFLP-PCR) assay, due to ability of their PCR products digestion by *NciI* and *PvuII* restriction enzymes, respectively. Indeed, G848A nucleotide variation in *SPATA16* gene causes disruption of *NciI* site in this location. Thus, a partial PCR product (635 bp) of this gene encompassing G848A could not be cut to produce 283 and 352 bp fragments. Similarly, mutation of G198A region in *PICK1* gene disrupts one of two *PvuII* restriction sites located in this 548 bp PCR product. Thus, G198A mutation produces two bands after *PvuII* cut, lack of which could cause production of three bands after *PvuII* digestion. In this study, we did not evaluate the other mutations in these two genes.

Following identification of three exons (5, 6 and 7) deletion in one of the affected Iranian individual (G9) which was previously reported by Elinati et al. (16), and due to the history of infertility in his family, blood samples of several volunteer family members were obtained and the target of interest was analyzed in their DNA samples.

For detection of *DPY19L2* deletion, a multiplex PCR assay was performed for exons 1, 5, 6, 7, 11 and 22 of this gene, together with a part of *β-ACTIN* or PROTAMIN 1 genes, as internal control using specific primers (Table 2). Lack of amplification for all or some *DPY19L2* exons indicates respectively total or partial deletion of this gene in the studied cases. To confirm complete deletion of this gene, specific breakpoint “a” amplification was performed in the samples with lack of amplification for all *DPY19L2* exons. 

**Table 2:** List of primers used for polymerase chain reaction and real time PCR analysis


	Genes	Amplified sequences	Primer sequence (5'→3')	Annealing temperature (°C)	Product length (bp)

Conventional PCR	*β-ACTIN*	-	F: CGTGACATTAAGGAGAAGCTGTGC	55	375
			R: CTCAGGAGGAGCAATGATCTTGAT		
	*DPY19L2*	Exon 1	F: GGCCAACTTCTTTCTACTCGGAC	65	504
			R: GACCCAGCTCCACCATACTCCTT		
		Exon 4	F: CAAAATAGCGAGAAGTGATTAG	54	414
	R: TTCTACTCAACTATAAGGATACAC				
		Exon 5	F: AGCTTCATCCATGTCACTAT	60	432
	R: AGCCTTCTCAGAAAACTATTTT				
		Exon 6	F: GGGTAAATAATTAAACACAGCA	57	462
	R: AAACAACAGAATAAAAGGGAT				
		Exon 7	F: AATTTATACGTACACTTTTTAGAATTA	55	420
	R: ATTTAAACATTTCAATCAACATGC				
		Exon 8	F: TGGACATGGTAGTTAATTGCTG	55	371
	R: TCCCAAAGTGCTGAATTGAA				
		Exon 11	F: AACCTCCTCAAGTGACTTAG	53	516
	R: TTGGCCAAGAGTCATT				
		Exon 22	F: GTGTCTGTTATTAAAGCTTGTG	59	313
	R: ATTGTCTCTAGACAGCAATACAT				
	Break point “a”	-	F: ATGCCATGTTGCCTGCT	62	1700
			R: TCTTCTGGGAAAGGTATTATCGTAG		
	*SPATA16*	Exon 4	F: AATTCTTTGCCATTGTCATATC	58	635
			R: GGTCAAGCGCATTTCTATTAC		
	*PICK1*	Exon 13	F: TGGGCTGCCATCCATGATC	66	568
			R: GCTCCCAGGCTCCGTCCTC		
	*PROTAMINE1*	-	F: CCCCTGGCATCTATAACAGGCCGC	60	530
			R: TCAAGAACAAGGAGAGAAGAGTGG		
Real-time PCR	*β-ACTIN*	-	F: AGATGCGTTGTTACAGGAAG	60	92
			R: TGTGTGGACTTGGGAGAG		
	*DPY19L2*	-	F: GACCCAGCTCCACCATACTCCTT	60	144
			R: TTCCATCTCCTCCTCTACCTCCG		


### Quantitative assessment of mutated *DPY19L2* alleles

**Fig.1:**
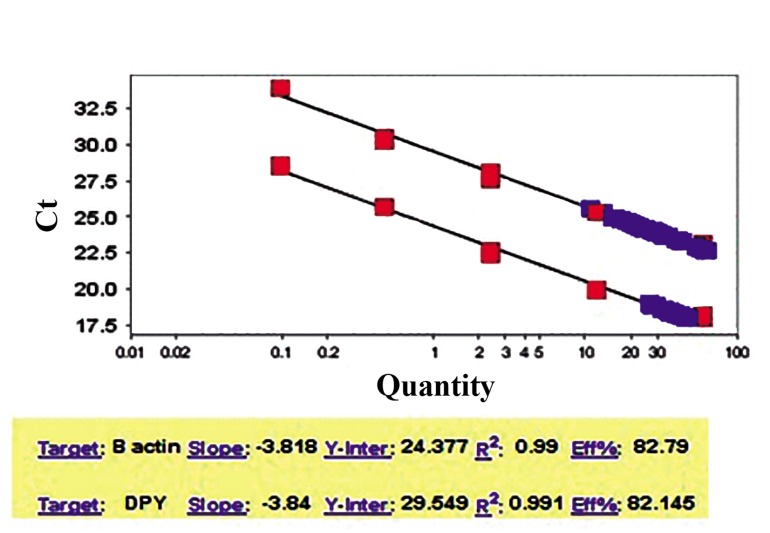
A standard threshold cycle (Ct) curve was drawn to calculate the allele quantities of *DPY19L2* and *β-ACTIN* for individuals who are suspected to be carrier for pathogenic allele of *DPY19L2*. As described in materials and methods, a standard Ct curve was drawn using different amounts of DNA from a fertile donor (60, 12, 2.5, 0.5 and 0.1 ng, red squares) through qPCR. Then, the quantity of the target gene (*DPY19L2*, lower curve) and the reference gene (*β-ACTIN*, upper curve) of each tested sample, for individuals who were suspected carriers of the pathogenic allele of *DPY19L2*, was calculated based on their Ct on the standard curve. The primer efficiency for both genes was almost similar. Meanwhile, regression coefficients (R2) and the slope of Ct curves were mostly equal (approximately 0.99, and-3.8 respectively).

qPCR was implemented by two alternative methods, to analyze the genotyping of *DPY19L2* gene for the family members of three cases (G8, 14 and 21) in terms of homo/hemizygosity deletion or normal state of *DPY19L2* gene. Specific *DPY19L2* and *β-ACTIN* primer pairs were designed to quantify both the target and reference genes (Table 2). Of note, primer efficiencies for target gene (*DPY19L2*) and reference gene (*β-ACTIN*) were almost equal (Fig .1).

In the first method, samples were quantified absolutely, using a control blood sample obtained from a healthy fertile donor, who voluntarily participated in this study. After genomic DNA extraction by standard salting out procedure, 60 ng of standard genomic DNA was used as a template for further serial dilution preparations. Different amounts of DNA (60, 12, 2.5, 0.5, 0.1 ng) from this fertile donor were used as template in each PCR reaction in three set of PCR to draw a standard threshold cycle (Ct) curve (red squares shown in the Fig.1). Then, 60 ng of sample tests were subjected to PCR reactions (blue squares shown in the Fig.1). The quantity of the target gene (*DPY19L2*, lower curve shown in the Fig.1) and the reference gene (*β-ACTIN*, upper curve shown in the Fig.1) of each subject was calculated based on their Ct in the standard curve which was drawn with different amounts of DNA from the fertile (control) sample in ABI step one plus real-time PCR system (Life Technologies, CA, USA). Proportion of PCR products of *DPY19L2* to *β-ACTIN* quantities was considered for further analyses. This proportion for fertile was considered between 0.8-1, for carrier and patient cases was approximately 0.5 (ranged 0.3-0.7) and 0 respectively, as reported earlier (18). Additionally, to assess the accuracy of this method, equal volume of DNA extracts from blood samples taken from the fertile individual (control sample) and a patient with globozospermia (G14) were mixed and the resulting mixture was used as a heterozygous (hetero) sample.

In the second method, conventional relative quantification (RQ, using 2^-ΔΔCt^ equation) method was used with the same samples, utilizing 60 ng of DNA templates to quantify PCR-products of *DPY19L2* relative to *β-ACTIN*. In this study, RQ level was considered 0.8-1 for normal cases, while this level was approximately 0.5 (ranged 0.3-0.7) and 0 in carrier and patients respectively, as previously reported (18). All PCR reactions contained 5 µl SYBR Green (TaKaRa, Japan), 0.2 µl Rox and 5 µM of each specific primer (0.2 µl) for *DPY19L2* or for *β-ACTIN* (0.5 µl) in a 10 µl final volume of PCR reaction.

## Results

### Clinical characteristics of the patients with globozoospermia

Clinical parameters of the patients who participated in this study are depicted in the Table 3. Analyses showed lower sperm motility of the patients, compared to the highlighted standard criteria by World Health Organization (WHO). Regarding the round-headed shape of the sperms, in this study, ICSI technique was used to obtain successful fertilization culminated in three healthy births (Table 3). In this survey, three pedigree members that suffered from globozospermia were further studied. 

**Table 3:** Clinical parameters of patients with globozoospermia


Patient	Consanguinity of the parents	Sperm parameters				ICSI attempts and results
		Round-headed sperm(%)	Volume (mL)	Sperm concentration (10^6^/mL)	Progressive motility (%)	Number of ICSI (ET cycles)	Clinical pregnancy (Abortion)	Live delivery (Sexuality)

G1	Non-familial	100	3	80	10	2	No (-)	-
							No (-)	
G2	NA	100	3	80	30	1	No (-)	-
G3	NA	100	4	64	15	ND	-	-
G4	Familial	100	3	20	5	ND	-	-
G5	Familial	100	3.5	40	2	ND	-	-
G6	Familial	100	4	66	10	ND	-	-
G7	Non-familial	100	1	65	25	ND	-	-
G8	Non-familial	100	4	66	10	1	Yes (-)	Ongoing
G9	Familial	100	4	50	20	1	No (-)	-
G10	Familial	100	2.5	40	20	3	No (-)	1 Singleton (Girl)
							yes (+)	
							Yes (-)	
G12	Non-familial	100	1.5	70	20	1	Yes (-)	1 Singleton (Boy)
G13	Non-familial	100	0.5	67	25	2	No (-)	1 Singleton (Girl)
							Yes (-)	
G14	Familial	100	1	2	25	ND	-	-
G15	Non-familial	100	2	74	0	ND	-	-
G16	Non-familial	100	3	30	15	1	No (-)	-
						(1)	No (-)	
G18	NA	100	4	80	10	1	No (-)	-
						(1)	No (-)	
G19	NA	98	4	80	0	1	Yes (-)	Ongoing
G20	Familial	100	3	40	15	1	No (-)	-
						(1)	No (-)	
G21	Non-familial	100	3.1	10	5	ND	-	-
							No (-)	
G22	Non-familial	100	6.7	60	35	2	No (-)	-
G23	Non-familial	100	2.1	10	5	ND	-	-
G24	Familial	100	2	18	5	1	No (-)	-
G25	Familial	96	2.9	90	40	1	No (-)	-
G26	Familial	100	2.3	40	10	ND	-	-
G27	Familial	100	1	40	15	1	No (-)	-
						(1)	No (-)	
G28	Familial	98	2.3	28	10	ND	-	-
G29	Familial	100	3	45	40	1	No (-)	-


Two samples of G11 and G17 were lost, thus they were deleted. ET; Freeze-thawed embryo transfer, ICSI; Intra-cytoplasmic sperm inseminaton, NA; Not assigned, ND; Not done, +; Stands for successful pregnancy, -; Stands for abortion, and ^*^; Globo 23 is single and not married.

### Mutational analysis in *SPATA16* and *PICK1* genes

**Fig.2:**
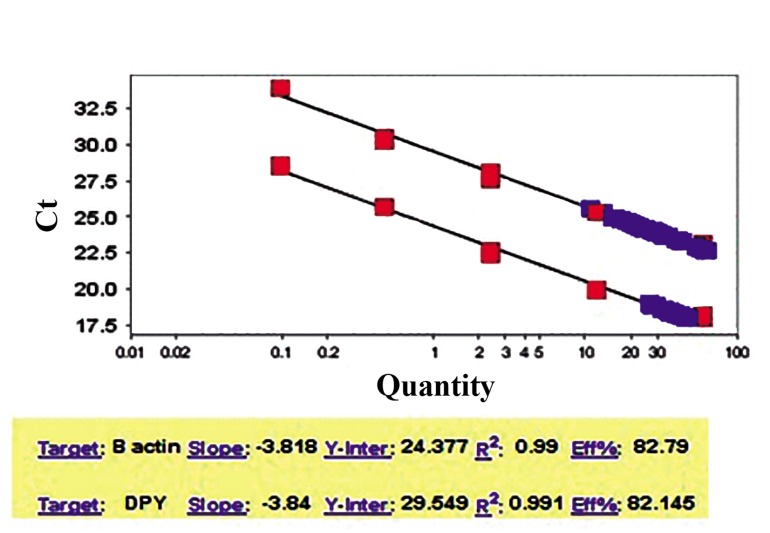
Assessment of missense mutations of G848A in the exon 4 of *SPATA16* (left panel) and G198A in the exon 13 of *PICK1* (right panel) genes using *NciI* and *PvuII* restriction endonuclease enzymes, respectively. No mutation was observed due to complete digestion of amplified fragments as described in materials and methods. M; 50 bp DNA ladder.

In this study, 27 cases with globozoospermia and 30 fertile men as control group were analyzed for detection of nucleotide variation (Table 1). In our first screening, regarding that digestion of the PCR products resulted in similar pattern to the fertile cases (data not shown), we did identify missense mutations of neither G848A in exon 4 of *SPATA16* gene (Fig .2, left panel) nor G198A in exon 13 of *PICK1* gene in the studied cases (Fig .2, right panel).

### Analysis of *DPY19L2* deletion in the cases with globozoospermia

We have previously reported that *DPY19L2* gene deletion leads to globozoospermia (16). In this study, further to 14 (out of 18) individuals who had shown some deletion in *DPY19L2* gene, six (out of nine) new cases with globozoospermia, missed the entire length of *DPY19L2* gene (G21, 22, 23, 24, 26 and 27, Table 1). One of these six individuals was unrelated (G24), while the remaining individuals were originated from two different pedigrees (Table 1). 

**Fig.3:**
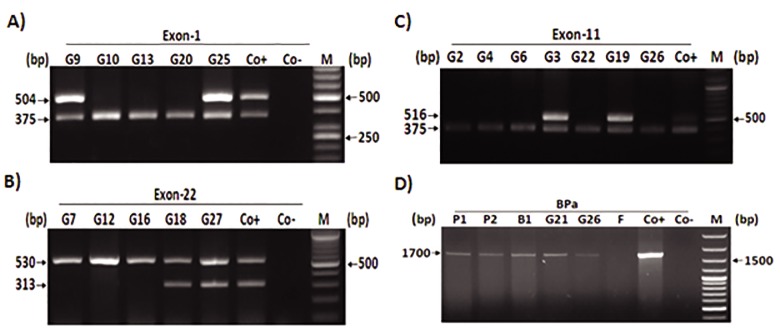
Analysis of *DPY19L2* gene deletion in exons 1, 11 and 22 as well as identification of breakpoint “a” (BPa) in a number of in- dividuals with globozoospermia (G#). Multiplex PCR products of A. Exon 1(504 bp, upper band), B. Multiplex PCR products of exon 22 of *DPY19L2* gene (313 bp, lower band) and part of *PROTAMINE1* gene (530 bp, upper band). Co+ or positive control in A, B and C is a fertile specimen and Coor negative control is no template sample, C. Exon 11 (516 bp, upper band) of *DPY19L2* gene together with a part of *β-ACTIN* gene (375 bp, lower band) and D. PCR analysis of BPa. P1 and P2 are parents of globozoospermia patient (G21) and B1 is his fertile brother and negative control is a fertile specimen, F, and positive control is a case with globozoospermia, which has been confirmed to have BPa. M; 50 bp DNA ladder in panel A and 100 bp DNA ladder for the rest of the panels and PCR; Polymerase chain reaction.

This experiment was carried out by several multiplex PCR on exon 1 (Fig .3A), exon 11 (Fig .3B) and exon 22 of *DPY19L2* gene (Fig .3C) with a part of *β-ACTIN* or *PROTAMINE1* gene. Two new individuals (G25 and 28), with partially globozoospermia demonstrations, showed a wild-type condition for *DPY19L2* gene (Table 1). Furthermore, data indicated the presence of breakpoint “a” (BPa) in most of the new cases (five out of six) with entire *DPY19L2* gene deletion (Fig .3D, Fig respective lanes for G21 and 26).

**Fig.4:**
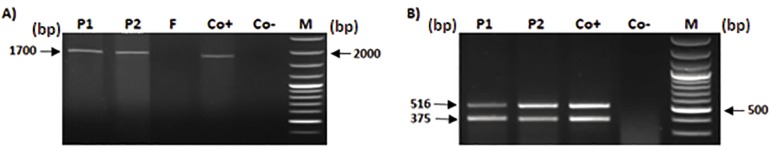
Detection of exon 11 and BPa in parents of one case with globozoospermia (G8). A. Amplification of BPa implied hemizygosity state for *DPY19L2* gene in the parents. DNA sample of fertile individual (F) was not amplified for BPa as expected, Co+; Globozoospermia who previously proved to have BPa, , P1, P2; Parents of G8, Co-; No template sample and B. Multiplex PCR products for exon 11 of *DPY19L2* gene (516 bp, upper band) together with a part of *β-ACTIN* gene (375 bp, lower band), Co+; Sample from a fertile man, Co-; No DNA template, M; 100 bp DNA ladder and P1, P2; Parents of G8.

Moreover, *DPY19L2* gene hemizygosity (complete deletion of one *DPY19L2* gene allele) was evaluated in parents of one case (G8), who has previously been recognized to suffer from deletion of entire length of *DPY19L2* gene (16). Here we confirmed hemizygosity of *DPY19L2* for both parents of G8, by amplification of BPa (Fig .4A) and exon 11 (Fig .4B). Of note that other siblings of this family were fertile.

### Evaluation of familial globozoospermia

**Fig.5:**
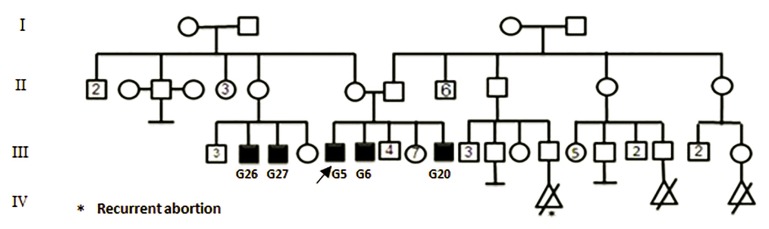
Pedigree of one case with globozoospermia (G5) and repeated pregnancy loss. There is more consanguineous marriage in this fam- ily but for simplicity detailed data are not depicted in the pedigree. Polymerase chain reaction (PCR) analysis showed G26, 27, 5, 6 and 20 suffering from globozoospermia due to complete deletion of *DPY19L2* gene. II2, III11 and III15 are infertile individuals with performing no genetic analysis. The inset numbers which are shown in the squares/circles represent the numbers of healthy (fertile) siblings who were not shown in this pedigree.

**Fig.6:**
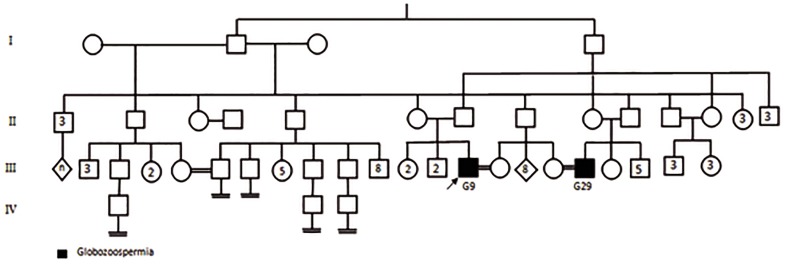
Consanguineous pedigree of the G9 family, with partial deletion of *DPY19L2* gene. Only one cousin (G29) who was also suffering from globozoospermia had deletion of exons 5, 6 and 7. The inset numbers which are shown in the squares/circles represent the numbers of healthy (fertile) siblings who were not shown in this pedigree. n; The sexuality and numbers of siblings were not determined.

In this experiment study, two cases (G5 and 9) were selected for sibling analysis. As shown in the Figure 5, the genetic pedigree belongs to family of G5 (with the history of reproductive failure and miscarriage) revealed that all of five members (G5, 6, 20, 26, and 27) had globozoospermia associated with complete deletion of *DPY19L2* gene. We have previously demonstrated (16) a partial deletion of *DPY19L2* including exons 5, 6 and 7 in one case (G9, Table 1). Due to infertility history of his family (Fig .6) and access to DNA samples of all family members, multiplex PCR of the aforementioned exons was performed. Curiously, we determined similar mutations pattern of *DPY19L2* gene to G9 patient, in the cousin with complete globozoospermia (G29). Indeed, detection of exons 4 and 8 by PCR confirmed this partial deletion (data not shown). 

### *DPY19L2* genotyping analysis

**Fig.7:**
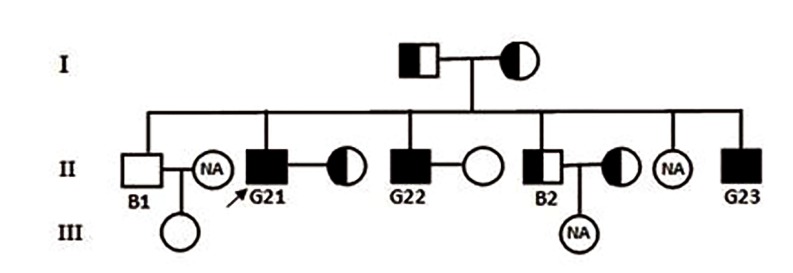
Consanguineous pedigree of G21 with complete deletion of *DPY19L2* gene in BPa. *DPY19L2* genotyping analysis in this pedigree indicated that B2 and wives of G21 and B2 are hemizygote. Three pedigree members (B1 wife, G21 sister and B2 daughter) did not par- ticipate in the analysis and their zygousity state remained undefined (NA)

To set a reliable method for homo/hemizygosity state of *DPY19L2* deletion, we performed qPCR based genotyping analysis for family members of one case who showed whole *DPY19L2* gene deletion, G21. The *DPY19L2* deletion consan- guineous pedigree for G21 patient is shown in the Figure 7. Data analyses demonstrated whole *DPY19L2* gene deletion of one allele for the G21 parents. Quantity proportion values of *DPY19L2* to *β-ACTIN* for carriers of *DPY19L2* deletion were almost between 0.40.6, while in normal cases, it ranged between 0.8-1 based on two calculated methods (quantities ratio and 2-∆∆Ct ratio presented in the Table 3). In addition, no amplification plot was detected for G21, 22 and 23 indicating lack of the mentioned gene in these subjects. In addition, detection of BPa in hemizygote individuals of the family confirmed the outcomes of qPCR assay (Fig .3D, Fig where P1 and P2 are parents of G21 patient, B1 is the hemizygote fertile brother. G21 is proband person) (Table 4).

**Table 4:** DPY19L2 genotype achieved by quantitative real-time PCR for the family members of Globo 21 and Globo 22


Subject	Quantities ratio	2^-∆∆Ct^ ratio

Father (P1)	0.522924	0.4
Mother (P2)	0.408959	0.4
Brother-1 (B1)^*^	1.006982	1
B1 sibling	0.957183	0.9
Brother-2 (B2)^*^	0.499332	0.4
B2 wife	0.414017	0.4
Globo22 wife	1.037303	1
Globo21 wife	0.488268	0.4


^*^; B1 and B2 are 2 fertile patient’s (Globo21) brothers, Ct; Threshold cycle, and PCR; Polymerase chain reaction.

**Table 5:** 


Subject	Quantities ratio	2^-∆∆Ct^ ratio

Globo 14 father	0.58377	0.5
Globo 14 mother	0.40623	0.4
Globo 8 father (P1)	0.47476	0.5
Globo 8 mother (P2)	0.424836	0.4
Hetero	0.560374	0.4
Control-1	0.973175	0.9
Control-2	1.381863	1.3

Ct; Threshold cycle and PCR; Polymerase chain reaction.

To extend the application of previously suggest- ed method (quantities ratio) for identification of gene homo/hemizygosity at different individuals, we performed further analyses on the G8 and 14 patients’ parents, besides of the hemizygote sam- ple (hetero) as notified in materials and methods. Data affirmed the hemizygote status of the parents and hetero case by two alternatively implicated calculation methods (Table 5).

## Discussion

In the recent years, there have been an increasing amounts of literatures proposing the molecular mechanisms of globozoospermia (7,9,13,15,16,19,21). Our previous studies have described *DPY19L2* gene as a basic factor required for development of normal acrosome biogenesis. Partial or complete deletion of the *DPY19L2* gene is pivotal factor in globozoospermia (16). 

Therefore, we investigated complete deletion of *DPY19L2* gene effects to reaffirm the potential association of *DPY19L2* gene and globozoospermia. In addition, deletion of this gene was evaluated in the family members of three globozoospermic individuals. Thus, deletion analysis of *DPY19L2* gene (12q14.2) was carried out in three exons 1, 11, 22 of *DPY19L2* gene, using multiplex PCR, compared to *β-ACTIN* or *PROTAMINE1* genes as internal controls. Briefly, all of three assessed exons of *DPY19L2* gene (1,11,22) were missed in 20 out of 27 cases (74%) suggesting total absence of *DPY19L2* gene in these cases. 

It should be noted that identification of total deletion of *DPY19L2* gene with BPa in 18 cases, out of 27, has previously been reported by Elinati et al. (16). Overall, six out of nine new individuals showed complete deletion of *DPY19L2* gene, five of whom carried BPa and the remaining may have unknown BP. Also, one new patient (G29) harbored a partial deletion of this gene and two others (G25 and G28) with partial globozoospermia had two wild type alleles. Previous studies have also demonstrated molecular mutations in *DPY19L2* gene (19,21). Deletion of the *DPY19L2* gene is a common genomic rearrangement that occurs due to LCRs flanking the gene by NAHR. Concurrent with the cases, family members of three globozoospermic patients were investigated in this study. In this regard, two pedigrees (G5 and 9 pedigrees) from different geographically accommodation regions, similar ethnicity and high rate of consanguineous marriages showed the history of reproductive failure due to globozoospermia. Regarding high tincidence of this rare abnormality among tribal races, diagnosis of carrier individuals could help them, in terms of genetic management, for future family planning. 

Several studies have previously detected heterozygosity of the other genes, like SMN1 and DYSTROPHIN, through quantitative real-time PCR based on comparative Ct method (18,22,23). In this article, we identified the carriers in one pedigree (G21 pedigree) by this method and also proposed a modified method, quantities ratio. Thus, we designed qPCR assay for family members of G21. Analyses were performed based on proportion of *DPY19L2* to *β-ACTIN* quantities. After providing the standard curve based on serial diluted DNA samples of a fertile man, quantities of the reference and target gene were estimated. Quantitative analysis of *DPY19L2* gene for G21 family members led us to identify individuals with hemizygosity at this gene. We determined that parents with a quantity ratio ranging between 0.4-0.6 are carrier. One of the fertile brothers (B2) as well as partners of G21 and B2, were hemizygote for deletion of *DPY19L2* gene. Quantity ratio for normal cases, consisting one of the fertile brother, the grand daughter and partner of G22, were ranging from 0.95-1.3. These results were similar to previously reported threshold cycle method verifying our conclusion to determine the individuals with no gene deletion or carriers (18,22). Considering non-consanguinity of parents, the incidence of the abnormality in this family could be attributed to their accommodation in the same geographical area. To validate our calculation method on the allele hemizygosity, we extended experiments on the G8 and G14 patients’ parents who kindly accepted to participate voluntarily in this survey. 

These findings are in agreement with previous studies, indicating a strong relationship between *DPY19L2* gene and globozoospermia. However, molecular cause of few cases remains yet unclear, requiring further investigations to identify genetic defect(s) in the other gene(s) affecting globozoospermia. 

Regarding the other genes, in the present study mutation screening of the *SPATA16* and *PICK1* genes were also carried out on 27 cases with globozoospermia and 30 fertile men. Our data revealed that *PICK1* and *SPATA16* genes were intact in all of studied individuals. 

## Conclusion

Our result revealed that qPCR analysis can be used for genotyping of *DPY19L2* deletion and this may help genetic consolers in family planning. In future, it might also help prevent occurrence of this syndrome in carrier families through pre-implantation genetic diagnosis, especially in ethnic community with high consanguineous marriages. 
